# Long-term Outcomes After Surgical Resection of Pancreatic Metastases from Renal Clear-Cell Carcinoma

**DOI:** 10.1245/s10434-021-09649-w

**Published:** 2021-02-11

**Authors:** Giuseppe Malleo, Roberto Salvia, Laura Maggino, Giovanni Marchegiani, Michael D’Angelica, Ronald DeMatteo, Peter Kingham, Alessandra Pulvirenti, Elisabetta Sereni, William R. Jarnagin, Claudio Bassi, Peter J. Allen, Giovanni Butturini

**Affiliations:** 1 Unit of General and Pancreatic Surgery–DSCOMI University of Verona, Verona, Italy; 2 Unit of General and Pancreatic Surgery, Department of Surgery and Oncology, University of Verona Hospital Trust, Verona, Italy; 3 The Hepato-Biliary and Pancreatic Unit, Memorial Sloan Kettering Cancer Center, New York, NY USA; 4 Present Address: Department of Surgery, University of Pennsylvania, Perelman School of Medicine, Philadelphia, PA USA; 5 Present Address: Department of Surgery, Duke University, Durham, NC USA; 6 Present Address: Unit of Pancreatic Surgery, Pederzoli Hospital, Peschiera del Garda, Italy

## Abstract

**Background:**

Pancreatic metastases (PM) from renal cell carcinoma (RCC) are uncommon. We herein describe the long-term outcomes associated with pancreatectomy at two academic institutions, with a specific focus on 10-year survival.

**Methods:**

This investigation was limited to patients undergoing pancreatectomy for PM between 2000 and 2008 at the University of Verona and Memorial Sloan Kettering Cancer Center, allowing a potential for 10 years of surveillance. The probabilities of further RCC recurrence and RCC-related death were estimated using a competing risk analysis (method of Fine and Gray) to account for patients who died of other causes during follow-up.

**Results:**

The study population consisted of 69 patients, mostly with isolated metachronous PM (77%). The median interval from nephrectomy to pancreatic metastasectomy was 109 months, whereas the median post-pancreatectomy follow-up was 141 months. The 10-year cumulative incidence of new RCC recurrence was 62.7%. In the adjusted analysis, the relative risk of repeated recurrence was significantly higher in PM synchronous to the primary RCC (sHR = 1.27) and in patients receiving extended pancreatectomy (sHR = 3.05). The 10-year cumulative incidence of disease-specific death was 25.5%. The only variable with an influence on disease-specific death was the recurrence-free interval following metastasectomy (sHR = 0.98). In patients with repeated recurrence, the 10-year cumulative incidence of RCC-related death was 35.4%.

**Conclusion:**

In a selected group of patients followed for a median of 141 months and mostly with isolated metachronous PM, resection was associated with a high possibility of long-term disease control in surgically fit patients with metastases confined to the pancreas.

The pancreas is an uncommon metastatic site for renal clear-cell carcinoma (RCC). Pancreatic metastases (PM) are mostly metachronous to the primary tumor and are typically identified after a disease-free interval of many years.^1^ The current treatment options include surgical resection^1^–^14^ or newly introduced biologic agents directed at vascular endothelial growth factor receptor (VEGF-R), tyrosine kinases (TK), or mammalian target of rapamycin (mTOR).^15^–^17^ Because of the infrequency of PM, it has been particularly challenging to accrue clinical data supporting one strategy over the other, with no randomized trials of resection versus targeted therapy having been conducted to date. When resection is undertaken, the extent depends on the size and the number of metastases. Options include parenchyma-sparing procedures for small lesions, formal resections, as well as total pancreatectomy and pancreatectomy with contiguous organ resection for multifocal or bulky lesions. The available observational studies and systematic reviews of patients undergoing surgical resection report 5-year survival projections from 33% to 72% at median follow-up times of 24–91 months.^1^–^14^ Hypothesizing that the 10-year survival rate might be a more informative outcome measure, we analyzed long-term oncologic outcomes of patients who had the potential for 10 years of follow-up following metastasectomy at two large academic institutions. Unlike previous studies that estimated recurrence and survival using the standard method of Kaplan–Meier, we employed a competing risk analysis to account for death by other causes during follow-up.

## Methods

### Study Design

The study was approved by the local Institutional Review Board (PAD-R, 1101 CESC). The electronic databases at the University of Verona and Memorial Sloan Kettering Cancer Center were queried to identify patients with histologically confirmed PM from RCC who underwent pancreatic resection. The search was limited to patients treated from January 2000 up to December 2008, to allow a potential for 10 years of follow-up. Demographic, clinical, surgical, and pathologic details were captured and analyzed retrospectively. Per institutional practices, pancreatectomy (either for synchronous or metachronous PM) was typically indicated when the pancreas was the only metastatic site. Extended pancreatectomy with contiguous organ resection was performed for bulky lesions with extrapancreatic infiltration. Atypical resections were considered only for PM < 20 mm, even in the instance of multifocal lesions. Postoperative complications (pancreatic fistula, delayed gastric emptying, and post-pancreatectomy hemorrhage) were defined according to the current criteria.^18^ Operative mortality was defined as death within 90 days of operation. Follow-up was generally carried out every 6 months for the first 2 years and at yearly intervals thereafter. Cross-sectional imaging, clinical examination and routine blood tests were carried out at follow-up visits. Repeated recurrences in the pancreas were treated at the authors’ institutions, whereas in the instance of repeated extra-pancreatic recurrences, patients were referred to other specialists and treated according to the current standards. Details and outcomes of surgical, interventional procedures, or medical therapies for new pancreatic and extra-pancreatic recurrence were tracked in our databases and analyzed. All patients had updated information on further RCC recurrence and vital status at the time of data lock.

### Statistical Analysis

The distribution of continuous variables is reported as medians and range, the Mann**–**Whitney *U* test was used to compare medians. Categorical variables are presented as numbers and percentages; the chi-squared test was used for statistical comparison. Fisher**’**s exact test was used when appropriate. All tests were two-sided. For recurrence and survival analysis, data were censored at the date of last follow-up/disease recurrence or last follow-up/death by disease, respectively. Because of the slow-growing nature of RCC and the long follow-up period, a competing risk analysis was performed. This estimates the marginal probability of next disease recurrence or death by disease in the presence of a competing event (death from other causes). The cumulative incidence functions (CIF) for the outcomes of interest were plotted and the 5- and 10-year cumulative incidences, stratified by an array of clinically relevant variables, were calculated. Pairwise comparison of CIF across strata of these variables was performed using Gray’s test. Next, the effect of covariates on the CIF for the event of interest was estimated using the subdistribution hazard regression model according to Fine and Gray. The subdistribution function estimates the hazard of failing from the event of interest at time *t* based on the risk set that remains at time *t* after accounting for all previously occurring event types, which includes competing events. The resulting subdistribution hazard rates (sHR) denote the relative change in the instantaneous *rate* of the occurrence of the primary event in subjects who have not experienced it.^19^^,^^20^ An extension for time-dependent covariates was used to account for variables for which the proportional hazard assumption did not hold. The most parsimonious adjusted models were selected according to the Akaike information criterion (AIC). *P*-values are presented with sHR and 95% confidence intervals. Statistical significance was determined by a *p*-value of < 0.05. Data were analyzed using the R.3.6.2 software, packages ‘survival’ and ‘cmprsk’ (Foundation for Statistical Computing, Vienna, Austria; https://www.r-project.org).

## Results

### Demographics and Surgical Results

The study population consisted of 69 patients (43 from the University of Verona and 26 from Memorial Sloan Kettering Cancer Center). There were 41 males (59.4%) and 28 females (40.6%), and the median age at the time of PM presentation was 66 years (range 24–84). In nine patients (13%) the PM was synchronous to the primary RCC, and a multifocal pattern was observed in 10 patients (14.5%). In 53 patients (76.8%) the pancreas was the first metastatic site following nephrectomy, and in seven patients (10.1%) the first metastatic site had been extra-pancreatic (lung in two cases, liver, thyroid, adrenal, bone, and skin). These seven patients had previously received specific treatment and had no evidence of extra-pancreatic disease at the time of PM identification. The median interval from primary RCC resection to pancreatic metastasectomy was 109 months (range 0–294). Table 1 shows the operative procedures performed in the study population. The rate of postoperative morbidity was 34.8% (24 patients). Fifteen patients developed a clinically relevant pancreatic fistula (21.7%), five experienced delayed gastric emptying (7.2%), and three patients (4.3%) had a post-pancreatectomy hemorrhage. Two patients died of complications 13 and 60 days postoperatively (2.9%). The median hospital stay was 10 days (range 4–123). Ten patients (14.5%) were pN1, and there were four R1 resections (5.8%).

**Table 1:** Overview of the procedures performed in patients with pancreatic metastases from RCC

Procedure	*N*
*Procedures for pancreatic metastases synchronous to the RCC*	
Distal pancreatectomy and radical nephrectomy	7
Distal pancreatectomy, radical nephrectomy and gastrectomy	1
Pancreatoduodenectomy and radical nephrectomy	1
*Procedures for metachronous pancreatic metastases*	
Distal pancreatectomy	30
Pancreatoduodenectomy	8
Total pancreatectomy	4
Middle-segment pancreatectomy	4
Atypical resection	3
Distal pancreatectomy and kidney enucleation	2
Pancreatoduodenectomy and kidney enucleation	1
Distal pancreatectomy and left colectomy	1
Distal pancreatectomy and left adrenalectomy	1
Pancreatoduodenectomy and right adrenalectomy	1
Pancreatoduodenectomy and subtotal gastrectomy	1
Pancreatoduodenectomy and right colectomy	1
Atypical resection and right adrenalectomy	1
Distal pancreatectomy and head enucleation	1
Middle-segment pancreatectomy and head enucleation	1

### Recurrence and Survival Analysis

Survival analyses were conducted in 67 patients, after excluding those who had died of postoperative complications. The median postoperative follow-up was 141 months (range 5–269) in the overall population and 165 months (range 110–269) in censored cases. At the end of follow-up, 13 patients (19.4%) were alive and disease-free, 20 patients (29.9%) were alive with a new RCC recurrence, 23 patients (34.3%) died of disease, and 11 patients (16.4%) died of other causes. Because the absolute percentage of deaths from other causes was in excess of 10%, the adoption of a competing risk model was justified (20).

Overall, 48 patients (71.6%) developed a new RCC recurrence after pancreatic metastasectomy. The pancreas, lung, and kidney were the predominant recurrence sites. Thirty patients (61%) had a single recurrence, whereas 18 patients (39%) had multiple recurrences. Table 2 summarizes the post-pancreatectomy recurrence pattern and the associated treatment strategies. Remarkably, eight of 48 patients with new recurrence (16%) underwent iterative pancreatic resections, including five completion pancreatectomies. Five patients who developed new RCC recurrence died of other causes.

**Table 2:** Pattern of recurrence and associated treatments after pancreatectomy for metastatic RCC

Recurrence site	Resection	CT	IT	TKI	Cyberknife	No treatment
*First recurrence after pancreatic metastasectomy* (*n* = 48)						
Pancreas (*n* = 13)	6	3	0	1	0	3
Lung (*n* = 11)	4	3	0	2	0	2
Kidney (*n* = 9)	5	0	0	2	0	2
Liver (*n* = 4)	1	0	0	2	0	1
RPLN (*n* = 4)	2	0	0	2	0	0
Bone (*n* = 2)	2	0	0	0	0	0
Adrenal (*n* = 1)	0	1	0	0	0	0
Brain (*n* = 1)	0	0	0	0	1	0
Thyroid (*n* = 1)	0	0	1	0	0	0
Unknown site (*n* = 2)	–	–	–	–	–	–
*Second recurrence after pancreatic metastasectomy (n = 19)*						
Pancreas (*n* = 5)	1	1	1	1	0	1
RPLN (*n* = 3)	0	0	1	2	0	0
Kidney (*n* = 2)	1	0	1	0	0	0
Bone (*n* = 2)	0	0	0	2	0	0
Adrenal (*n* = 1)	0	0	0	0	0	1
Brain (*n* = 1)	0	0	0	1	0	0
Ileopsoas (*n* = 1)	0	0	0	1	0	0
Lung (*n* = 1)	0	0	1	0	0	0
Thyroid (*n* = 1)	1	0	0	0	0	0
Duodenum (*n* = 1)	0	0	0	1	0	0
Unknown site (*n* = 1)	–	–	–	–	–	–
*Third recurrence after pancreatic metastasectomy (n = 8)*						
Pancreas (*n* = 2)	1	0	0	1	0	0
RPLN(*n* = 2)	0	0	0	2	0	0
Bone (*n* = 1)	0	0	0	1	0	0
Brain (*n* = 1)	0	0	0	0	1	0
Liver (*n* = 1)	0	0	0	0	0	1
Oesophagus (*n* = 1)	0	0	0	0	0	1

*RPLN* retroperitoneal lymph nodes, *CT* chemotherapy, *IT* immunotherapy, *TKI* tyrosine kinase inhibitors.

**Fig. 1:**
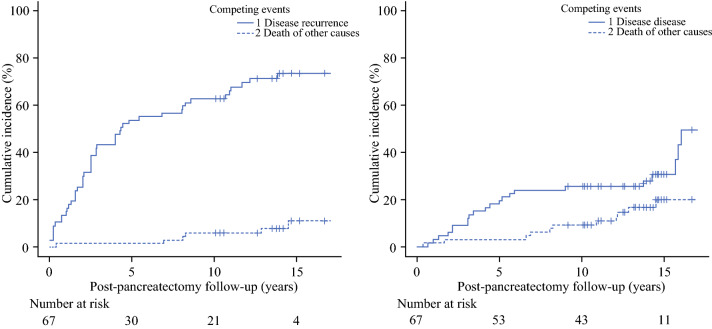
Predicted cumulative incidence functions for (**a**) disease recurrence and (**b**) death by disease following pancreatectomy for metastatic RCC

The CIF for post-pancreatectomy recurrence is shown in Fig. 1a: the 5-year and 10-year cumulative incidence rates were 53.7% and 62.7%, respectively. The 5-year and 10-year cumulative incidence stratified by clinically relevant variables is shown in Table 3a. Results of regression analysis are summarized in Table 4. Extended pancreatectomy was associated with a 3-fold relative incidence of new recurrent disease compared with standard pancreatectomy (adjusted sHR 3.05, 95% CI 1.72–5.40, *p* = 0.001). Notably, most patients receiving extended resection recurred at distant sites (12 versus 6 events). The relative incidence of repeated recurrence was 27% higher for PM synchronous to the primary RCC (adjusted sHR 1.27, 95% CI 0.98–2.84, *p* = 0.057).

**Table 3:** Cumulative incidence functions for (a) disease recurrence and (b) death by disease stratified by each level of clinically relevant covariates. Death from other causes was handled as a competing event in each analysis (data not shown)

Covariates	(a) Disease recurrence			(b) Disease-specific death		
	5-year, %	10-year, %	Comparison of CIF	5-year, %	10-year, %	Comparison of CIF
	(95% CI)	(95% CI)	*p* value*	(95% CI)	(95% CI)	*p* value*
*Overall*	53.7 (41.0–64.9)	62.7 (49.8–73.1)	NA	19.4 (10.9–29.7)	25.4 (15.6–36.3)	NA
*Sex*						
Female	60.0 (39.7–76.4)	67.9 (46.4–82.2)	0.56	17.9 (6.3–34.1)	21.4 (8.5–38.2)	0.21
Male	48.7 (32.1–63.4)	59.0 (41.6–72.8)		20.5 (9.5–34.4)	28.2 (15.1–42.9)	
*Age*						
<65 years	60.0 (39.8–75.3)	60.0 (39.8–75.3)	0.87	26.7 (12.4–43.3)	33.3 (17.2–50.4)	0.27
≥65 years	48.6 (31.6–63.7)	64.9 (39.8–75.3)		13.5 (4.9–26.6)	18.9 (8.2–33.0)	
*Pancreatic metastasis*						
Metachronous	50.0 (12.5–79.4)	50.0 (12.5–79.4)	0.53	18.6 (9.9–29.5)	25.4 (15.1–37.1)	0.55
Synchronous with RCC	54.2 (40.6–66.0)	64.4 (50.6–75.3)		25.0 (3.0–57.9)	25.0 (3.0–57.9)	
*Metastatic pattern*						
Single	50.9 (37.1–63.1)	59.6 (45.6–71.2)	0.18	14.0 (6.5–24.4)	21.1 (11.5–32.5)	0.77
Multifocal	70.0 (28.2–90.4)	80.0 (32.6–95.7)		50.0 (16.3–76.8)	50.0 (16.3–76.8)	
*Type of pancreatic resection*						
Formal	53.4 (39.7–65.4)	62.1 (48.1–73.3)	0.61	20.7 (11.3–32.0)	25.9 (15.4–37.7)	0.89
Atypical	55.6 (17.5–82.0)	66.7 (23.5–89.3)		11.1 (5.0–40.9)	22.2 (2.7–53.4)	
*Extended pancreatectomy*						
No	50.0 (35.6–62.8)	59.6 (44.8–71.7)	0.19	17.3 (8.5–28.8)	23.1 (12.7–35.3)	0.42
Yes	66.7 (35.3–85.4)	73.3 (40.7–89.9)		26.7 (7.7–50.6)	33.3 (11.4–57.4)	
*R-status*						
R0	55.6 (42.3–66.9)	63.5 (50.1–74.2)	0.74	20.6 (11.6–31.4)	27.0 (16.7–38.4)	0.72
R1	25.0 (3.4–71.4)	50.0 (2.3–88.1)		0	0	
*N-status*						
N0	50.9 (37.1–63.1)	59.6 (45.6–71.2)	0.107	17.5 (9.0–28.5)	24.6 (14.3–36.4)	0.16
N1	70.0 (28.2–90.4)	80.0 (32.7–95.6)		30.0 (6.2–59.3)	30.0 (6.2–59.3)	
*New disease recurrence*						
No	NA	NA	NA	–	–	0.007**
Single recurrence				26.7 (12.4–43.3)	33.3 (17.2–50.4)	0.436†
Multiple recurrence				27.8 (9.7–49.5)	38.9 (16.8–60.7)	

^*^Gray’s test for comparison of cumulative incidence functions (CIF)

**Overall *p*-value, Wald test

^†^Pairwise comparison of single versus multiple recurrence excluding patients who remained disease-free

**Table 4:** Subdistribution hazard regression model for disease recurrence (death from other causes was handled as the competing event)

Covariate	Crude				Adjusted			
	Disease recurrence		Death by other causes		Disease recurrence		Death by other causes	
	sHR	*p *value	sHR	*p *value	sHR	*p *value	sHR	*p *value
	(95%CI)		(95%CI)		(95%CI)		(95%CI)	
*Sex*								
Female	Reference	0.56	Reference	0.23	–	–	Reference	0.18
Male	0.84 (0.48–1.47)		3.68 (0.42–31.62)				4.32 (0.50–37.04)	
*Age*								
<65 years	Reference	0.89	Reference	0.74	–	–	–	–
≥65 years	0.96 (0.54–1.68)		0.77 (0.16–3.68)					
*Time from nephrectomy*	0.99 (0.99–1.01)	0.75	1.00 (0.99–1.01)	0.52				
*Pancreatic metastasis*								
Metachronous	Reference	0.63	Reference	0.64	Reference	0.057	–	–
Synchronous with RCC	1.34 (0.57–2.84)		1.63 (0.20–13–05)		1.27 (0.98–2.84)			
*Metastatic pattern*								
Single	Reference	0.29	–*	–	–	–	–	–
Multifocal	1.58 (0.67–3.71)							
*Type of pancreatic resection*								
Formal	Reference	0.62	Reference	0.88	–	–	–	–
Atypical	0.81 (0.36–1.83)		0.86 (0.12–5.96)					
*Extended pancreatectomy*								
No	Reference	0.28	Reference	0.76	Reference	0.001	–	–
Yes	1.51 (0.71–3.23)		0.71 (0.08–5.87)		3.05 (1.72–5.40)			
*R-status*								
R0	Reference	0.68	–*	–	–	–	–	–
R1	0.82 (0.33–2.05)							
*N-status*								
N0	Reference	0.072	–*	–	–	–	–*	–
N1	1.76 (0.94–3.28)							

sHR indicates subdistribution hazard rate

^*^R-status and N-status showed quasi-complete separation and were removed from the final model

The CIF for disease-specific death is plotted in Fig. 1b, the 5-year and 10-year cumulative incidence rates were 19.4% and 25.5%, respectively. Table 3b shows the 5-year and 10-year cumulative incidence stratified by clinically relevant variables. Table 5 outlines the results of regression analysis. The relative incidence of RCC-related death decreased by 2% per recurrence-free time unit (adjusted sHR 0.98, 95% CI 0.96–0.99, *p* = 0.001). Being older (cutoff 65 years) increased the probability of dying from other causes (adjusted sHR 5.71, 95%CI 1.36–24.68, *p* = 0.017).

**Table 5:** Subdistribution hazard regression model for RCC-specific death (death from other causes was handled as the competing event)

Covariate	Crude				Adjusted			
	Death by disease		Death by other causes		Death by disease		Death by other causes	
	sHR	*p*-value	sHR	*p* value	sHR	*p* value	sHR	*p* value
	(95%CI)		(95%CI)		(95%CI)		(95%CI)	
*Sex*								
Female	Reference	0.2	Reference	0.73	–	–	–	–
Male	1.76 (0.73–4.22)		1.24 (0.36–4.22)					
*Age*								
<65 years	Reference	0.18	Reference	0.5	–	–	Reference	0.017
≥65 years	0.56 (0.24–1.30)		1.51 (0.44–5.14)				5.81 (1.36–24.68)	
*Time from nephrectomy*	1.00 (0.99–1.01)	0.94	1.00 (0.99–1.01)	0.2	–	–	–	–
*Pancreatic metastasis*								
Metachronous	Reference	0.62	Reference	0.81	–	–	–	–
Synchronous with RCC	1.33 (0.42–4.25)		0.72 (0.11–5.39)					
*Metastatic pattern*								
Single	Reference	0.06	–*	–	Reference	0.07	–	–
Multifocal	2.56 (0.93–7.04)				2.49 (0.91–6.83)			
*Type of pancreatic resection*								
Formal	Reference	0.98	Reference	0.69	–	–	–	–
Atypical	0.99 (0.33–2.60)		0.67 (0.99–4.60)					
*Extended pancreatectomy*								
No	Reference	0.84	Reference	0.71	–	–	–	–
Yes	1.07 (0.51–2.25)		0.75 (0.16–3.35)					
*R-status*								
R0	Reference	0.62	Reference	0.68	–	–	–	–
R1	0.64 (0.11–3.64)		1.45 (0.24–8.70)					
*N-status*								
N0	Reference	0.055	–*	–	–	–	–*	–
N1	2.00 (0.98–4.07)							
*Disease-free interval*†	0.97 (0.96–0.99)	0.001	1.00 (0.99–1.01)	0.29	0.98 (0.96–0.99)	0.001	–	–

*sHR* subdistribution hazard rate

^*^N-status showed quasi-complete separation and was removed from the final model

^†^Handled as a time-dependent covariate

Another model limited to the subgroup of patients with new disease recurrence following pancreatectomy showed that neither the number (single versus multiple, sHR 0.85, 95% CI 0.36–2.0, *p* = 0.72) nor the site of new recurrence (repeated pancreatic recurrence versus extra-pancreatic new recurrence, sHR 0.36, 95% CI 0.1–1.24, *p* = 0.11) had a significant effect on disease-specific survival. The 5- and 10-year cumulative incidence rates of disease-specific death in patients with any repeated recurrence following pancreatectomy were 27.1% and 35.4%, respectively. In patients who remained disease-free, the 5- and 10-year cumulative incidence of death by other causes was 3% and 9%, respectively.

## Discussion

Pancreatic metastasectomy is only occasionally performed because metastatic lesions to the pancreas are uncommon, accounting for 2% of all pancreatic neoplasms. In the absence of prospective trials, indications for the interventional approach include the primary cancer type, primary cancer site control, anatomic respectability, and patient tolerance for pancreatectomy.^12^^,^^13^ In the present study we investigated the long-term outcomes of 69 patients who underwent pancreatectomy for metastatic RCC at two large academic institutions. This slow-growing tumor has a peculiar tropism for the pancreas, in that the ratio of metastases increases in a time-dependent manner, reaching a plateau well beyond 10 years from nephrectomy.^21^–^23^ Such a long tumor latency may be due to different mutational landscapes and angiogenesis mechanisms relative to metastases at typical sites (lung, bone, liver, and lymph nodes), that mostly occur within 5 years.^16^ The available data from patients undergoing surgical resection for PM report favorable outcomes, with 5-year survival rates up to 72%.^1^–^14^ Assuming 10-year survival is a more pertinent outcome measure, given that the disease course is often indolent, we enrolled patients up to 2008 and made sure that all the subjects still living had an adequately updated recurrence and vital information at the time of data lock. This resulted in a median follow-up of 141 months in the overall population and of 165 months in censored cases. To account for the long observation time and the median patient age at the time of metastasectomy, a competing risk analysis was employed. This is a type of survival analysis estimating the probability of new disease recurrence or death from RCC in the presence of a possible competing event (death from other causes) that hinders the observation of the event of interest.^19^^,^^20^ Traditional methods used to describe the survival process (i.e., the method of Kaplan–Meier) assume noninformative censoring and are not designed to accommodate the competing nature of multiple causes to the same event, producing inaccurate estimates of cause-specific probabilities. Hence, direct comparison between our results and traditional overall survival estimates reported in previous literature is not necessarily applicable.

In this study 71.6% of patients developed a new post-pancreatectomy recurrence, with a 10-year cumulative incidence of 62.7%. The pattern of recurrence was heterogeneous as nearly two thirds of patients experienced a single event while the remaining had multiple new recurrences. Remarkably, the pancreas was the most frequent site of repeated tumor relapse. Among the 19 patients with new recurrence in the pancreatic remnant (27.5%), eight underwent iterative resection (five completion pancreatectomies and three enucleations) because the second recurrence was isolated, and the patients were surgically fit.

In the adjusted subdivision hazard regression, the relative risk of repeated recurrence was higher in PM synchronous to the primary RCC, with marginal significance (*p* = 0.057). The proportion of synchronous PM was 13%, a lower figure compared with collective series of unselected advanced patients.^24^ In fact, the combined resection of an initially metastatic RCC is indicated only if all tumor deposits are excised in the context of a single or oligo-metastatic resectable disease.^25^ In the more common instance of a metachronous presentation, PM occurred with the well-known latency following nephrectomy (median of 109 months). The probability of post-pancreatectomy recurrence was also increased in patients undergoing extended resections, although new relapses occurred mostly at distant sites and RCC-specific survival was comparable with standard pancreatectomy. This resonates with a recent study by Di Franco et al. favoring pancreatectomy with multivisceral resection for locally advanced PM, as happened in an impressive 43% of their caseload, because an aggressive surgical approach did not compromise overall survival.^9^

At the 10-year mark, the cumulative incidence of disease-specific death in the overall population was 25.5%. This figure increased to 35.4% in patients with recurrent disease, indicating that 64.6% were alive with new recurrence following pancreatectomy. Although the estimated cumulative incidence derived using the Kaplan–Meier approach is in general larger than estimates obtained when accounting for competing risk (with resulting lower survival rates), the present results seem to compare favorably with previous studies reporting 10-year outcomes. As an example, in a paper by Schwarz et al., the 10-year overall survival rate was 32% at a median follow-up of 91 months.^10^ These sharp differences in long-term survival projections may also depend on the number of censored cases before the time-mark of interest in each dataset. Notably, in our study no patient was censored before 110 months from metastasectomy.

The only variable with a significant influence on RCC-related death was the interval from PM resection to the next RCC recurrence (adjusted sHR of 0.98, *p* = 0.001). Neither the new relapse site (pancreatic or extra-pancreatic), nor the number of post-pancreatectomy recurrences (single or multiple) affected survival. Previous studies have described a connection between a long disease-free interval from initial nephrectomy to PM and improved survival.^6^^,^^9^ This was not found in the present analysis.

Our results may have certain implications for clinical decision-making. Following the introduction of targeted therapies, now used in all lines of metastatic RCC, it has been suggested that the need for pancreatic metastasectomy may be reduced or even obviated, because resection did not significantly prolong survival as compared with targeted therapies only.^17^ Conversely, other analyses have suggested that complete remission with biologic agents is limited, and that survival outcomes are better following local therapy, especially in the instance of isolated PM.^24^^,^^26^ The present study does not add comparative information, because most patients received resection before the introduction of biologic agents (sorafenib was approved for advanced RCC in 2005).^15^ However, in the absence of prospective trials and recommendations specific to PM, metastasectomy might be offered to surgically fit patients with isolated metastases, implementing first-line biologic therapy in subjects at high risk for postoperative morbidity.

Several major limitations apply to this analysis, including the retrospective nature, the small sample size, and the referral bias towards good surgical candidates with isolated PM. This may limit the application of our findings when considering the whole collective of patients presenting with metastatic disease. Furthermore, prognostic models validated to predict overall survival in metastatic RCC, including MSKCC and International Metastatic RCC Database Consortium (IMDC) criteria^27^^,^^28^ could not be applied because some biochemical parameters were lacking in both databases.

With these limitations in mind, the present study showed that resection of PM from RCC is associated with a 62.7% cumulative incidence of repeated recurrence and a 25.5% cumulative incidence of RCC-specific death at the 10-year mark, accounting for death by other causes as a competing event. Although the treatment of metastatic RCC is optimized on a case-by-case basis, it can be inferred that resection offers a high possibility of disease control in good surgical candidates with isolated PM.
